# Patient perceptions of disease burden and treatment of myasthenia gravis based on sentiment analysis of digital conversations

**DOI:** 10.1038/s41598-024-57825-1

**Published:** 2024-03-27

**Authors:** Ashley Anderson, Jacqueline Pesa, Zia Choudhry, Caroline Brethenoux, Patrick Furey, Louis Jackson, Liliana Gil Valleta, Laura Gonzalez Quijano, Alex Lorenzo

**Affiliations:** 1 Department of Neurology, Houston Methodist Hospital, Houston, TX USA; 2 Janssen Scientific Affairs, LLC, 1125 Trenton-Harbourton Rd., Titusville, NJ 08560 USA; 3 Janssen Pharmaceuticals, US Medical Affairs, Titusville, NJ USA; 4 CulturIntel DBA Human Dot Plus, Irving, TX USA; 5 Janssen Scientific Affairs, LLC, Horsham, PA USA

**Keywords:** Burden, Digital conversation, Myasthenia gravis, Patient perspective, Sentiment analysis, Social media, Neuroscience, Diseases, Neurology

## Abstract

Myasthenia gravis (MG) is a rare, autoimmune, antibody-mediated, neuromuscular disease. This study analyzed digital conversations about MG to explore unprovoked perspectives. Advanced search, data extraction, and artificial intelligence-powered algorithms were used to harvest, mine, and structure public domain digital conversations about MG from US Internet Protocol addresses (August 2021 to August 2022). Thematic analyses examined topics, mindsets, and sentiments/key drivers via natural language processing and text analytics. Findings were described by sex/gender and treatment experience with steroids or intravenous immunoglobulin (IVIg). The 13,234 conversations were extracted from message boards (51%), social media networks (22%), topical sites (21%), and blogs (6%). Sex/gender was confirmed as female in 5703 and male in 2781 conversations, and treatment experience was with steroids in 3255 and IVIg in 2106 conversations. Topics focused on diagnosis (29%), living with MG (28%), symptoms (24%), and treatment (19%). Within 3176 conversations about symptoms, eye problems (21%), facial muscle problems (18%), and fatigue (18%) were most commonly described. Negative sentiments about MG were expressed in 59% of conversations, with only 2% considered positive. Negative conversations were dominated by themes of impact on life (29%), misdiagnosis problems (27%), treatment issues (24%), and symptom severity (20%). Impact on life was a key driver of negativity in conversations by both men (27%) and women (34%), and treatment issues was a dominant theme in conversations by steroid-treated (29%) and IVIg-treated (31%) patients. Of 1382 conversations discussing treatment barriers, 36% focused on side effects, 33% on lack of efficacy, 21% on misdiagnosis, and 10% on cost/insurance. Side effects formed the main barrier in conversations by both steroid-treated and IVIg-treated patients. Capturing the patient voice via digital conversations reveals a high degree of concern related to burden of disease, misdiagnosis, and common MG treatments among those with MG, pointing to a need for treatment options that can improve quality of life.

## Introduction

Disease surveillance using “social media listening”, the process of analyzing online conversations to find mentions of a particular disease, provides a unique opportunity to gain an unprovoked perspective on patients’ sentiments toward a disease^1^. Social media platforms enable patients to create communities in which they can share their experiences and perceptions of their disease with other patients^2^. There is a growing interest in the potential of examining digital conversations through techniques such as big-data machine-learning, to provide insights into patient perspectives about their disease and associated treatments. Natural language processing is increasingly being used in healthcare research to analyze large volumes of social media posts about various diseases^3,4^. Themes such as symptoms, diagnostic journey, lived patient experiences, clinical and socioeconomic burden of disease, and treatment patterns and preferences can be identified easily via machine-learning techniques and quantified to reflect the frequency, and hence importance, of these discussions among patients^3–6^. The methodology has already been used to analyze attitudes toward depression among different ethnic groups^7,8^, barriers to breast cancer treatments^9^, and perceptions about suicidality among patients with epilepsy^10^.

An interesting application of natural language processing is to analyze social media posts on rare diseases such as myasthenia gravis (MG)—an autoimmune, antibody-mediated, neuromuscular disorder—to better understand the burden of disease from the patient perspective. Published estimates vary, but the Myasthenia Gravis Foundation of America (MGFA) estimates that MG affects between 14 and 20 people in every 100,000 in the US general population^11^. MG affects the structural and functional integrity of the postsynaptic neuromuscular junction, resulting in fluctuating bouts of weakness affecting the muscles that control the eyes, mouth, throat, and limbs^12^. Treatment is based largely on long-term immunosuppression or immunomodulation^13,14^, with newer therapeutic approaches shifting toward individualized, targeted, and selective immunological agents^15^. Corticosteroids remain the mainstay of MG treatment^16,17^, despite well-known safety and tolerability issues that limit their long-term use (e.g., weight gain, cardiac and gastrointestinal conditions, hypertension, glucose intolerance and diabetes, osteoporosis, and ophthalmological conditions)^15,18–20^. Intravenous immunoglobulin (IVIg) is commonly used to treat MG exacerbations and is also used as a maintenance therapy; however, it is associated with side effects, high cost, and inconvenience^15,21–23^.

Despite currently available treatments, patients with MG still experience a considerable disease burden^24,25^. The clinical manifestations of MG are numerous, including eye symptoms (e.g., ptosis, diplopia), facial weakness resulting in decreased facial expressiveness, jaw fatigue, dyspnea, dysphagia, dysarthria, neck and extremity weakness, and associated fatigue. In addition to experiencing symptoms from the condition, patients with MG are more likely to have medical complications, such as respiratory infections, arrhythmias, heart failure, and hypotension, along with certain comorbid conditions, including cardiovascular disease, hyperlipidemia, hypertension, diabetes, respiratory disorders, and concomitant autoimmune diseases^25^. The humanistic burden of MG extends to psychological comorbidities (i.e., depression, anxiety, and sleep disturbance) and reduced quality of life.

As with many other rare conditions, the impact of disease burden from the patient perspective has not been extensively evaluated and is poorly understood as a result^12^. To date, only a handful of small interview-based qualitative studies have examined patients’ feelings about their MG, its treatment, and the aspects they find most burdensome^26–29^. Findings from these studies indicate MG affects all aspects of patients’ lives, with a marked impact on patients’ emotional, social, and economic well-being arising from prevalent themes that include the unpredictable/uncertain nature of the disease, continual compromises and trade-offs, treatment inertia, and disconnect with healthcare providers (HCPs). As with all interview-based studies, findings may be limited in their generalizability and may be subject to certain recall and selection biases.

In this study, public domain digital conversations about MG were analyzed to describe mindsets and sentiments about the disease, its burden, and its treatments among men and women with MG and, more specifically, those treated with steroids and those receiving IVIg. Better awareness of patient perceptions of the burden of MG and its treatments will assist patient–HCP interactions and inform shared decision making.

## Methods

### Search strategy, data extraction, and collection

Advanced search, data extraction, and artificial intelligence (AI)-powered algorithms were used to harvest, mine, and structure public domain digital conversations and/or online comments (in English) among US adults who engaged in conversations about MG over a 12-month period (August 2021 to August 2022). Digital conversations were limited to those originating from US Internet Protocol addresses, mined by the topic of “myasthenia gravis”. Sources of conversations included message boards (online discussion sites where conversations take the form of posted messages, e.g., Reddit), topical sites (e.g., MGFA), social media networks (e.g., Facebook, Instagram, X [formerly Twitter]), content-sharing sites (e.g., TikTok, YouTube), blogs, and comments. Topical data were extracted and tagged by origin and user based on self-identification in the conversations or in public profiles using the CulturIntel™ methodology, which has been described previously^7,8^. Each unique relevant digital conversation was included in the analysis, forming a large unstructured dataset. Comments that appeared repeatedly through sharing or linking were only counted and analyzed once. Sex/gender-related and treatment-experience data were harvested and clustered where possible, based on user self-identification on open-source user profiles or in the unsolicited text and description within the analyzed conversations. All conversations were anonymized. Only online public domain data were collected and analyzed in an aggregated form, compliant with the General Data Protection Regulation. Datasets were deleted post-analysis.

### Data analysis

Digital conversations were tagged and sorted, and the most frequent discussion topics were identified via natural language processing and text analytics. Thematic analyses examined the most frequently discussed topics (including treatment barriers), sentiments (and key drivers of those sentiments), and overarching mindsets toward MG, and mapped underlying drivers and barriers to the stages of the MG patient journey. Analyses were human-assisted (by CulturIntel DBA HumanDot Plus, Irving, TX and the authors) and included repeated training, testing, and reviewing of the program output.

Conversations were categorized into four topics: diagnosis, treatment, living with MG, and symptoms. Of note, no categories or themes for these conversations were created a priori, rather, all categories and topical themes reported here were ‘naturally occurring’ within the data and identified by our analysis. Conversations about fatigue were described at a more granular level because of its complexity as a symptom in MG^30^ and the paucity of patient-centered data on this troubling manifestation.

Each conversation was categorized as positive, negative, or neutral in tone toward the subject of MG in a sentiment analysis using natural language processing, whereby AI algorithms were trained and validated to recognize these sentiments until an accuracy of 99.05% was reached. Following this broader classification, the most frequent drivers behind each sentiment were identified. Drivers of positive sentiments were considered reasons that patients are positive about MG (e.g., finding support, improving quality of life, getting access to treatment). Neutrality was defined as posing questions or sharing information about MG. Drivers of negative sentiments were classified as the reasons patients had negative feelings toward MG (e.g., symptom severity, impact on life, treatment issues, misdiagnosis).

Conversations about MG were classified into four possible overarching mindsets, using the following definitions: (1) uncertain (a sense of unpredictability and insecurity about the future, and a lack of confidence in coping with the challenges of living with MG); (2) pragmatic (being more practical toward the condition and focused on matters of fact rather than what could or should be); (3) struggling (a mental state in which the patient experiences ongoing difficulty coping with the challenges of MG); (4) indomitable (a strong and resilient attitude focused on overcoming obstacles and adapting, with an optimistic outlook toward the challenges of MG).

Each digital conversation was assigned to one of four possible stages of the patient journey in MG: suspected MG; getting diagnosed; managing; and ongoing assessment. Topics, mindsets, and treatment barriers were mapped to each stage of the journey.

Where possible, conversations were analyzed across patient segments of interest: sex/gender (male or female) and treatment type (steroids or IVIg). These characteristics were determined by scanning conversations for self-identification based on open user profile attributes and/or contextual text descriptions provided by the unsolicited digital conversations and comments analyzed.

### Statistical analyses

This was a descriptive study with categorical variables expressed as frequencies or percentages.

### Ethics approval and consent to participate

This study used publicly available, anonymized information and was exempt from institutional review board ethics approval.

## Results

### Digital conversations: search results

In total, 13,234 unique digital conversations about MG were extracted from message boards (51%), social media networks (22%), topical sites (21%), and blogs (6%) over 12 months (August 2021 to August 2022). Sex/gender could be determined through self-identification or public profiles in 64% (*n* = 8484) of conversations, with approximately two-thirds of conversations conducted by women (*n* = 5703) compared with one-third (*n* = 2781) by men (Table 1). MG treatment experience with steroids was identified among 3255 conversations, and treatment experience with IVIg among 2106 conversations. Key quotes from the digital conversations are shown in Table 2.

**Table 1:** Sentiment analysis about MG in digital conversations.

Digital conversations	Positive *n* (%)	Neutral *n* (%)	Negative *n* (%)
Overall (*N* = 13,234)	265 (2)	5161 (39)	7808 (59)
Sex/gender^a^			
Women (*n* = 5703)	114 (2)	2395 (42)	3194 (56)
Men (*n* = 2781)	28 (1)	1001 (36)	1752 (63)
Treatment			
Steroid (*n* = 3255)	0	1009 (31)	2246 (69)
IVIg (*n* = 2106)	21 (1)	821 (39)	1264 (60)

*N* and *n* indicate the number of digital conversations.

^a^Sex/gender could not be determined for 4750 digital conversations.

*IVIg* intravenous immunoglobulin, *MG* myasthenia gravis.

**Table 2:** Key quotes from digital conversations about MG.

Topic	Quote
Diagnosis difficulties	Do I have MG? … I do have one slightly droopy eyelid, so I did the look-up test, followed by the ice test. Looks to me like a pretty definitive MG outcome, but does anyone else have any insights to share?
	You shouldn’t have a doctor that you need to prove your disease to. You should have someone who is willing to help you and treat you with respect
	So, I will have to gather records and test info to carry with me since it is now clear the University certainly won’t diagnose me with a muscle disorder much less MG or protect and improve my quality of life
Symptoms	Does anyone else tremor with exertion…? My eyelids twitch when I squeeze my eyes closed, I have a rumble in my ears that’s similar, and even my tongue tremors when sticking it out. It’s a nightmare
	…[A] couple of years ago I started having these weird bouts of overwhelming fatigue on top of the usual fatigue, where it was impossible to do anything, or even think
Living with MG	Since April I've noticed some memory and concentration changes…I find my SHORT memory to be terrible and as [for] my concentration…
	Watching your life implode due to a disease is something only someone with that disease can understand…
Treatment	Treatment isn't helping much anymore. Neuro is thinking of IV medicine. Which ones have you taken, and how did you do with them? I'm getting scared
	I receive IVIg infusions every two weeks. I don't know why, but even with these treatments, I have been gradually doing worse over the past month or so…
	I don't feel that prednisone has helped at all
	I have reduced my Mestinon as it causes me severe twitching now. More side effects than benefits

*IV* intravenous, *IVIg* intravenous immunoglobulin, *MG* myasthenia gravis

### Conversation topics

**Figure 1:**
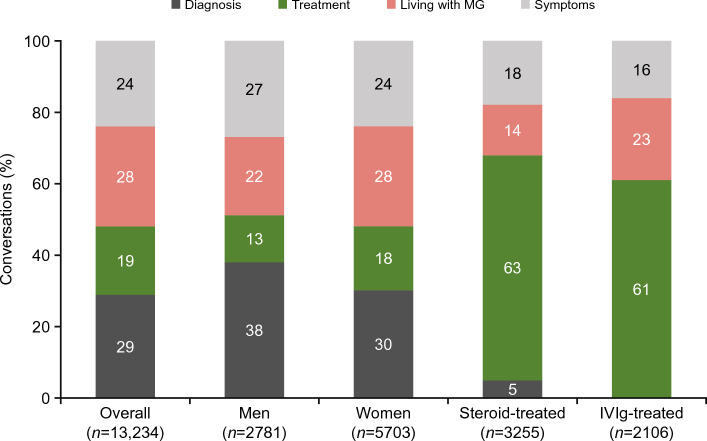
Topics discussed in digital conversations about MG. Note: *n* indicates the number of digital conversations; sex/gender could not be determined for 4750 digital conversations. *IVIg* intravenous immunoglobulin, *MG* myasthenia gravis.

Conversations were categorized into four topics: diagnosis (29%), living with MG (28%), symptoms (24%), and treatment (19%) (Fig. 1). Diagnosis was the focus of a relatively higher proportion of conversations by men compared to conversations by women (38% [1056/2781] and 30% [1710/5703], respectively). There was a relatively lower proportion of conversations by men than by women on the topics of living with MG (22% [612/2781] and 28% [1597/5703], respectively) and treatment (13% [361/2781] and 18% [1026/5703], respectively). More than 60% of all conversations of patients treated with steroids or IVIg focused on treatment.

Among the 3176 conversations about MG symptoms, eye problems (21%), fatigue (18%), facial muscle issues (18%), and weakness (17%) were most common. Respiratory issues, swallowing and speaking problems, muscle pain, and headaches were also discussed, albeit to a lesser extent (< 10% of conversations) (Additional file 1: Fig. S1). The profile of symptoms discussed in conversations was similar between women and men, as well as between those treated with steroids and those receiving IVIg.

**Figure 2:**
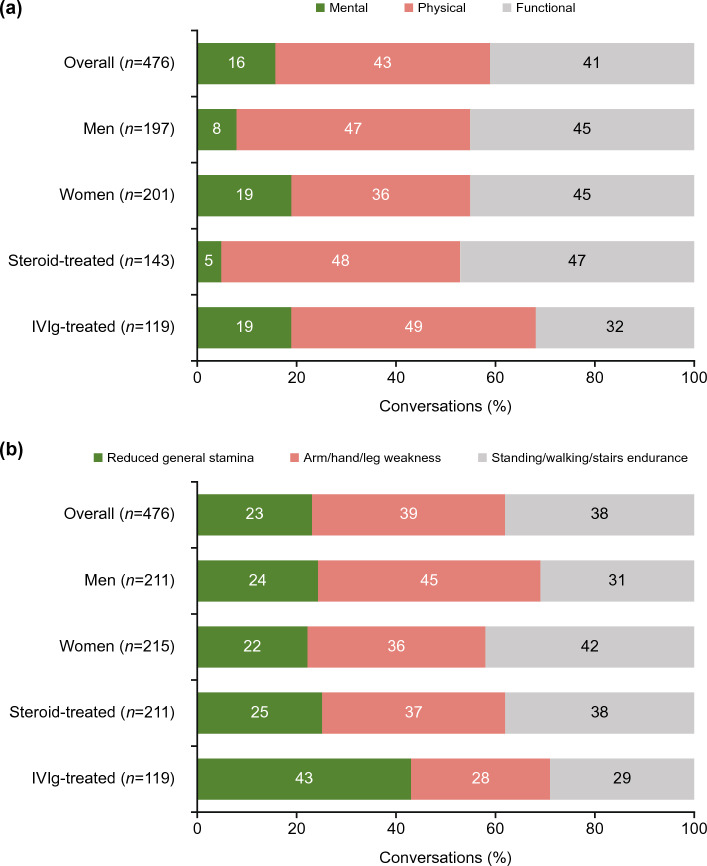
Impact of fatigue discussed by patients with MG in digital conversations: (**a**) contextual dimensions and (**b**) experiential dimensions. Note: *n* indicates the number of digital conversations. *IVIg* intravenous immunoglobulin, *MG* myasthenia gravis.

In total, 476 (4%) digital conversations discussed fatigue. The physical and functional impacts of fatigue were more commonly a topic of conversation than the mental impact (43%, 41%, and 16%, respectively) (Fig. 2a). Conversations focused on the impact of fatigue in terms of mobility/endurance (38%), weakness (39%), and stamina (23%) (Fig. 2b). Patterns were similar among conversations by men and women, although those by women more frequently focused on the mental impact of fatigue than those by men (19% [38/201] and 8% [16/197], respectively) (Fig. 2a). In addition, conversations about the mental impact of fatigue occurred in 19% (23/119) of IVIg-treated and 5% (7/143) of steroid-treated patients (Fig. 2a).

### Sentiment analysis

The majority of digital conversations (59%) expressed negative sentiments about MG, with only 2% of conversations considered to be positive in tone (Table 2). Conversations by men portrayed negative sentiments toward MG more frequently than those by women. None of the conversations by steroid-treated patients and only 1% of those by IVIg-treated patients expressed positive sentiments.

#### Key drivers of sentiments

**Figure 3:**
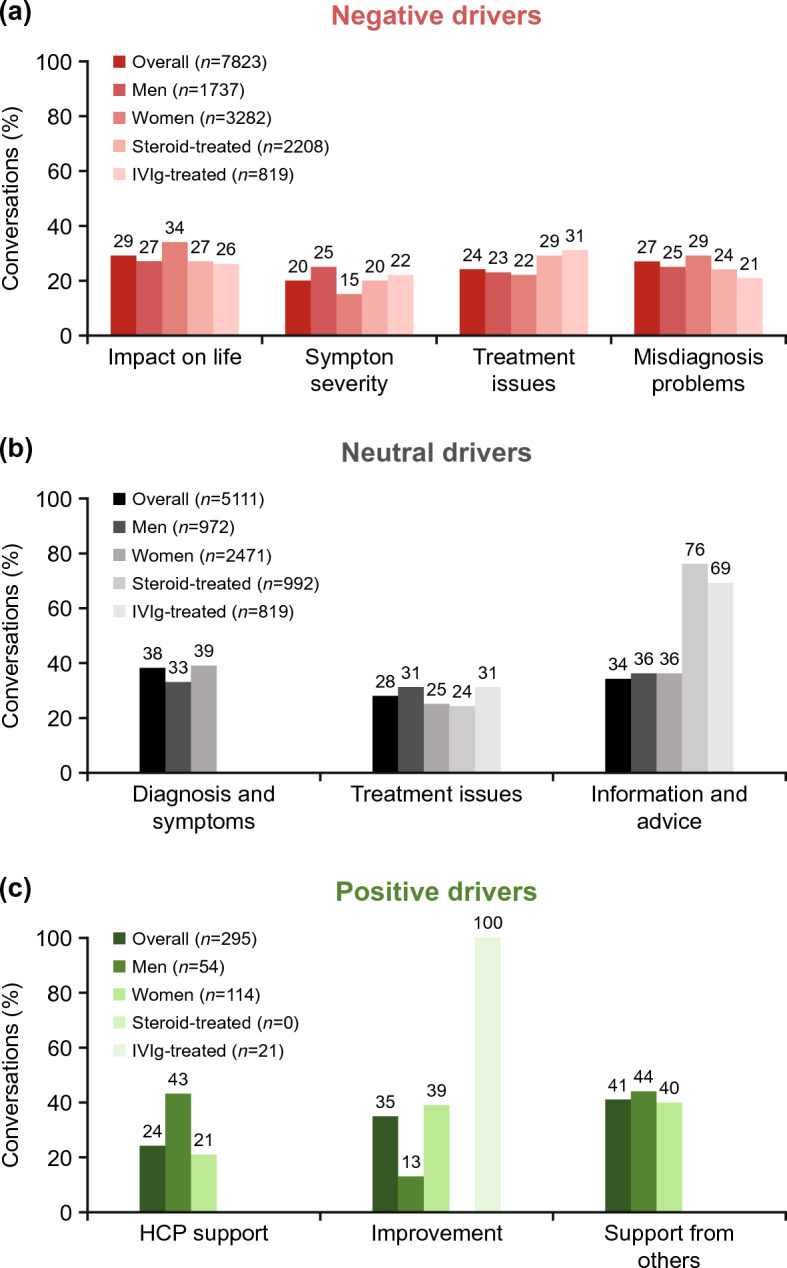
Negative (**a**), neutral (**b**), and positive (**c**) drivers for sentiments about MG expressed in digital conversations. Note: *n* indicates the number of digital conversations. *HCP* healthcare provider, *IVIg* intravenous immunoglobulin, *MG* myasthenia gravis.

Negative conversations were dominated by themes of impact on life (29%), misdiagnosis problems (27%), treatment issues (24%), and symptom severity (20%). Among conversations by both men and women, impact on life was the most common driver of negative sentiments toward MG, accounting for 27% (469/1737) of conversations by men and 34% (1116/3282) of conversations by women (Fig. 3). Symptom severity was also a key driver of negative sentiments in conversations by men but less so in conversations by women (25% [434/1737] and 15% [492/3282], respectively). Treatment issues was the dominant driver of negative sentiments in conversations by both steroid- and IVIg-treated patients, driving negativity to a similar extent in both groups (29% [640/2208] and 31% [254/819], respectively).

#### Treatment barriers

**Figure 4:**
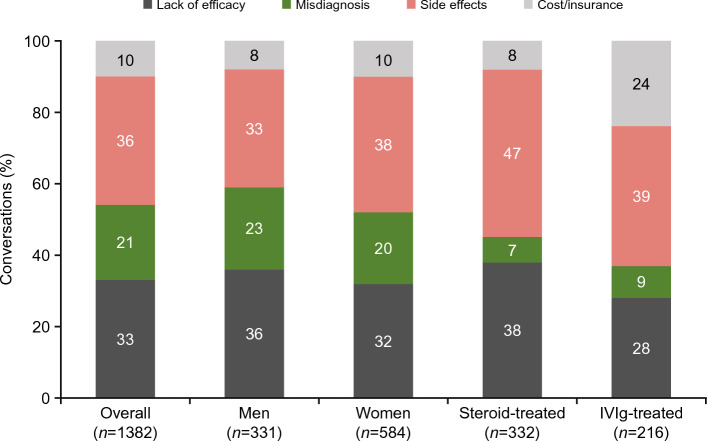
Treatment barriers among patients with MG identified in digital conversations. Note: *n* indicates the number of digital conversations. *IVIg* intravenous immunoglobulin, *MG* myasthenia gravis.

Of 1382 conversations about barriers to treatment, 36% focused on side effects, 33% on lack of efficacy, 21% on misdiagnosis, and 10% on cost/insurance (Fig. 4). There were no major differences in the proportions of conversations discussing each treatment barrier in conversations by men and women. However, differences in treatment barriers were observed when segmented by treatment type. The most discussed barrier to treatment in conversations by both steroid-treated and IVIg-treated patients was side effects (47% [156/332] and 39% [84/216], respectively), followed by lack of efficacy (38% [126/332] and 28% [60/216], respectively). Cost/insurance was cited as a barrier in 24% of conversations by IVIg-treated patients compared with only 8% of conversations by steroid-treated patients.

### Overarching mindset

Of 9824 conversations about MG in which the mindset could be determined, 39% were classified as uncertain, 32% as pragmatic, 21% as struggling, and 8% as indomitable (Additional file 1: Table S1). Most conversations by women were pragmatic (41%), while most conversations by men indicated uncertainty (42%). Differences in mindset were also seen in conversations categorized according to treatment type. Conversations by steroid-treated patients indicated a primarily uncertain mindset toward MG (41%), whereas conversations by IVIg-treated patients indicated a more pragmatic mindset (45%).

### Patient journey

**Figure 5:**
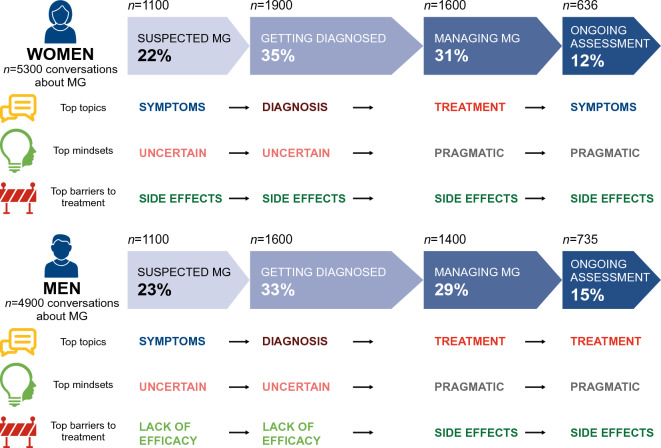
The MG journey from the patient perspective in women and men. *MG* myasthenia gravis. Note: *n* indicates the number of digital conversations.

As the MG patient journey progressed, so did the key focus of the digital conversations—in terms of topics, mindsets, and barriers to treatment (Fig. 5). Conversations by women were more uncertain initially, before becoming more pragmatic as MG treatment commenced; the most worrisome barrier throughout their journey was side effects. Among conversations by men, barriers focused initially on lack of efficacy, with challenges around side effects emerging and becoming more relevant as MG treatment started. Similarly, in conversations by steroid- and IVIg-treated patients, side effects became a greater focus of digital conversations as individuals moved through the patient journey.

## Discussion

This quantitative analysis of qualitative online public domain conversations examined unprovoked patient perspectives on disease and treatment burden in MG. Capturing the patient voice via digital conversations revealed a high degree of concern among patients, specifically related to symptoms, impact on life, misdiagnosis, and MG treatments. Only 2% of conversations were considered positive in tone. Key differences were observed between men and women in terms of sentiments and mindsets toward MG that could have implications for disease management, and a high degree of negativity was noted among patients with MG who had been treated with steroids or IVIg therapies, dominated by concerns surrounding side effects and lack of efficacy.

Side effects and lack of efficacy emerged as the leading barriers to treatment in digital conversations overall, with side effects being the most prominent treatment barrier following diagnosis for both men and women. A previous qualitative study in 14 patients with MG found that side effects of MG treatment were of particular concern, especially blood clots, infection/decreased immunity, weight gain, and diarrhea^31^. Side effects of prolonged steroid administration are well known and are ubiquitous in steroid-treated patients with MG^18^. As such, it was anticipated that the dominant treatment barrier in digital conversations by steroid-treated patients in our study would be side effects. In this analysis, the most frequent barrier to treatment was side effects, representing 47% and 39% of treatment conversations among steroid-treated patients and IVIg-treated patients, respectively.

Longitudinal study results from both a retrospective cohort study and a prospective observational study demonstrated that patients’ disease burden and symptoms are not well managed by conventional treatments^24,25^. Additionally, results from a qualitative study of patient perspectives conducted by interview demonstrated that obtaining symptom stability was a major treatment goal^26^. In this study, lack of efficacy was the second most prominent barrier to treatment overall, and the top barrier to treatment discussed by men in the early diagnostic stages of the disease. Real-world healthcare resource utilization study results revealed that the majority of patients with MG are treated with acetylcholinesterase inhibitors, corticosteroids, and/or non-steroidal immunosuppressive therapies per recommended treatment patterns^32^. However, in the present study, lack of efficacy was more prominent in digital conversations among steroid-treated patients (38%) than among IVIg-treated patients (28%), suggesting that patients with MG are concerned about steroid treatments and may be experiencing side effects. These findings are in line with previously published studies^18,33,34^. Furthermore, key quotes from digital conversations underscored the lack of efficacy as a treatment barrier, especially for steroid-treated patients.

Cost/insurance was a frequently discussed barrier (24% of conversations) among IVIg-treated patients. Unsurprisingly, given the low cost of steroid treatment, only 8% of conversations among steroid-treated patients focused on cost. Retrospective claims analyses indicate that costs for IVIg (or subcutaneous immunoglobulin) treatment account for the greatest proportion of total MG-related drug costs in the United States^32^. IVIg treatment for MG may include multiple infusions over 3–5 days^21^, and there is a dearth of studies conceptualizing patient perspectives on the time commitment associated with IVIg treatment. Studies in primary immunodeficiency that focus on the burden of IVIg from the patient perspective, rather than the HCP perspective, suggest IVIg treatment may be associated with a substantial patient burden in terms of time and commitment, due to factors such as organization and planning^35,36^.

As with other rare conditions, there is a paucity of information about the burden of MG from a patient perspective and limited understanding of patients’ unmet needs. A handful of previously published interview-based investigations into patient perspectives in MG identified a similar experience of living with MG as seen in the present study of digital conversations^26–29^. A study of 28 US patients found that approximately 90% experienced eye symptoms and fatigue, and all patients reported emotional, work, and financial impacts^26^. Eye problems and fatigue were also among the most discussed symptoms, reflecting a pattern of symptoms seen in real-world observational studies^7^. Fatigue is common in MG, particularly among women and those with more severe disease^30,37,38^, and is an important indicator of worsening quality of life^30,39^. While one domain of fatigue relates to the muscular disorder that impacts patients’ functional performance (peripheral fatigue), another is independent of muscle weakness, relating to a lack of energy and feelings of tiredness that interfere with patients’ mental and/or physical activities (central fatigue)^30,39^. In our study, physical and functional impacts of fatigue were discussed more commonly than the mental impact; however, women talked about the mental impact of fatigue more frequently than men (19% vs 8% of conversations). Reasons for this are not clear, although differences in social roles may have contributed. The mental impact of fatigue was also discussed more frequently in conversations by IVIg-treated patients compared with steroid-treated patients (19% vs 5%), possibly related to differences in disease severity.

Living with uncertainty, physical weakness, and significant changes made to daily life are experience themes of patients living with MG^28^. Themes of living with fluctuating symptoms, a continual need for assessment and adaptation, reluctance to alter treatments (leading to undertreatment), and a disconnect with HCPs, as well as feelings of anxiety, frustration, guilt, anger, loneliness, and depression driven by the burden of MG symptoms, social isolation, and a lack of support were all described in a study of 114 patients led by an international Patient Council^27^. As such, social and peer support, as well as developing psychological strategies to live with the impact of MG, are important factors for coping with an MG diagnosis, as found in a study of nine patients in Taiwan^29^. Social media, such as the outlets analyzed in the current study, can be an important source of support for patients to obtain knowledge about their disease, express feelings, and create a sense of community^2^. Overall, 8% of patients had an indomitable mindset, which may reflect a lack of control over MG or hope. These findings underscore the unmet need for treatments that better control symptoms and reduce MG exacerbations or crises, which can improve patients’ lives and may aid in shifting indomitable mindsets.

A plain language summary of this article is available in Additional file 2.

### Strengths and limitations

A strength of this study is that it included more than 13,000 unique digital conversations about MG. Beside the advantages that the methodology offers in terms of generating “big data”, analysis of digital conversations outside of HCP-led formal clinical or research environments also has the advantage of providing an unprovoked, candid view of the mindsets and attitudes of patients with MG, devoid of any “holding back” or reservation of opinion that may occur in conversation in the provider setting.

The study had some limitations. The presence of MG was based only on self-report in digital conversations and could not be confirmed by an HCP; this could be a source of potential bias. It is possible that the individuals who discuss MG on social media may not reflect the overall population with MG, which could limit the generalizability of the findings. Approximately two-thirds of the conversations analyzed in this study were conducted by self-identified women. This is attributed to information on gender available from profiles of social media users and is not reflective of sex differences in the prevalence of MG. However, more than 93% of Americans now regularly use the internet^40^, with 72% using some type of social media^41^. In addition, only digital conversations conducted in English and originating from US Internet Protocol addresses were collected.

## Conclusions

The study used advanced methodology to explore prevalent themes in MG from a patient perspective. The research technology provided a large dataset (> 13,000) of digital conversations about MG from US internet users with a self-reported diagnosis of MG. Capturing the patient voice via digital conversations revealed a high degree of concern among patients with MG related to burden of disease, misdiagnosis, treatment, and side effects. These findings highlight the limitations of currently available MG treatments and the need for improved options for people living with the disease that enhance symptom control with fewer and more manageable side effects. This information can be used to improve tailoring of management strategies to individual patient’s needs and priorities.

### Supplementary Information

Supplementary Information 1.

Supplementary Information 2.

## Data Availability

The data that support the findings of this study are available from CulturIntel DBA Human Dot Plus, but restrictions apply to the availability of these data, which were used under license for the current study, and so are not publicly available. Data are however available from the authors upon reasonable request and with permission of CulturIntel DBA Human Dot Plus.

## Abbreviations

AI: Artificial intelligence
HCP: Healthcare provider
IV: Intravenous
IVIg: Intravenous immunoglobulin
MG: Myasthenia gravis
MGFA: Myasthenia Gravis Foundation of America
