# Supplementary material for: Patient perceptions of disease burden and treatment of myasthenia gravis based on sentiment analysis of digital conversations
Source: Sci Rep. 2024 Mar 27;14:7271. doi: 10.1038/s41598-024-57825-1 (PMC10973330; doi:10.1038/s41598-024-57825-1)
Supplement: Supplementary file 1 — Supplementary Information 1. [file 41598_2024_57825_MOESM1_ESM.pdf]

Supplementary Information: Additional file 1

Figure S1 Profile of MG symptoms. [a] Other symptoms (overall) included respiratory issues (2.5%), muscle pain (1%), and headache (0.5%). Note: *n* indicates the number of digital conversations. *IVIg* intravenous immunoglobulin, *MG* myasthenia gravis

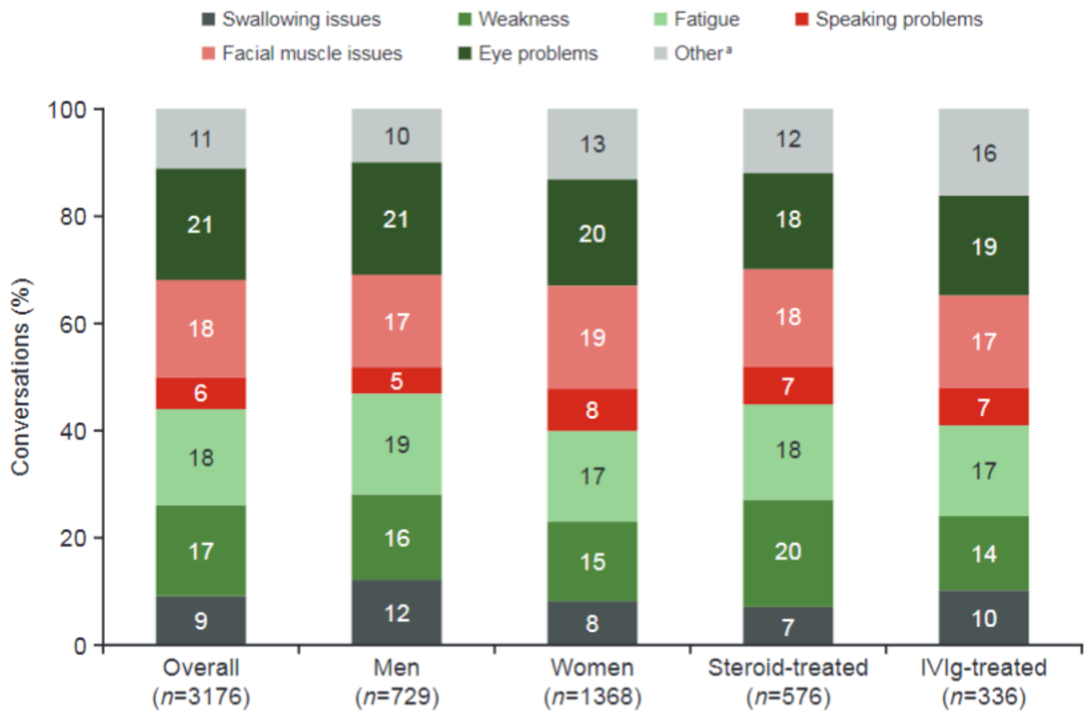

Table S1 Overarching mindsets of digital conversations towards MG by sex/gender and treatment

Conversations	Uncertain<br>***n* (%)**	Pragmatic<br>***n* (%)**	Struggling<br>***n* (%)**	Indomitable<br>***n* (%)**
Overall (*n*=9824)	3831 (39)	3144 (32)	2063 (21)	786 (8)
Sex/Gender				
Women (*n*=4227)	1353 (32)	1733 (41)	761 (18)	380 (9)
Men (*n*=1900)	798 (42)	570 (30)	399 (21)	133 (7)
Treatment				
Steroid (*n*=989)	405 (41)	307 (31)	277 (28)	0
IVIg (*n*=768)	207 (27)	346 (45)	192 (25)	23 (3)

Note: *n* indicates the number of digital conversations

IVIg intravenous immunoglobulin, *MG* myasthenia gravis
