# Supplementary material for: Patient perceptions of disease burden and treatment of myasthenia gravis based on sentiment analysis of digital conversations
Source: Sci Rep. 2024 Mar 27;14:7271. doi: 10.1038/s41598-024-57825-1 (PMC10973330; doi:10.1038/s41598-024-57825-1)
Supplement: Supplementary file 2 — Supplementary Information 2. [file 41598_2024_57825_MOESM2_ESM.pdf]

Supplementary Information: Additional file 2

PLAIN LANGUAGE SUMMARY

This study looked at digital conversations among patients with a rare, autoimmune disease called myasthenia gravis, which affects the muscles. Information was collected from online forums including message boards, social media networks, and blogs where people with myasthenia gravis talk about their experiences. The goal of this study was to understand myasthenia gravis from the perspective of a patient since most studies on the disease are based on information from doctors who care for people with myasthenia gravis. This study found that the most common themes were conversations about their diagnosed with myasthenia gravis, living with the disease, symptoms, and treatments. People with myasthenia gravis were most concerned with the burden of living with the disease, being misdiagnosed with a different condition, and treatment issues like side effects. The most commonly mentioned symptoms were eye problems and fatigue. Among the conversations where it was possible to determine the person's sex/gender, there were differences in the conversation topics between men and women across the patient journey. The concerns patients shared may mean that current treatment options are not meeting their needs for symptom control or their expectations for what constitutes an acceptable level and type of side effects. Therefore, people with myasthenia gravis need treatment options that can improve symptom control with fewer and more manageable side effects. Healthcare providers can use the findings from this study to tailor their care plan to their patients' individual needs and priorities.
